# Inhibition of nuclear factor-κB by 6-O-acetyl shanzhiside methyl ester protects brain against injury in a rat model of ischemia and reperfusion

**DOI:** 10.1186/1742-2094-7-55

**Published:** 2010-09-14

**Authors:** Wanglin Jiang, Shuping Zhang, Fenghua Fu, Haibo Zhu, Jian Hou

**Affiliations:** 1 Institute of Material Medica, Binzhou Medical University, Yantai, 264003, PR China; 2 State Key Laboratory of Long-acting and Targeting Drug Delivery Technologies (Luye Pharma Group Ltd.), Yantai, 264003, PR China; 3 School of Pharmacy, Yantai University, Yantai 264003, PR China

## Abstract

**Background:**

Recent studies have demonstrated an inflammatory response associated with the pathophysiology of cerebral ischemia. The beneficial effects of anti-inflammatory drugs in cerebral ischemia have been documented. When screening natural compounds for drug candidates in this category, we isolated 6-O-acetyl shanzhiside methyl ester (ND02), an iridoid glucoside compound, from the leaves of *Lamiophlomis rotata (Benth.) Kudo*. The objectives of this study were to determine the effects of ND02 on a cultured neuronal cell line, SH-SY5Y, in vitro, and on experimental ischemic stroke in vivo.

**Methods:**

For TNF-α-stimulated SH-SY5Y cell line experiments in vitro, SH-SY5Y cells were pre-incubated with ND02 (20 μM or 40 μM) for 30 min and then incubated with TNF-α (20 ng/ml) for 15 min. For in vivo experiments, rats were subjected to middle cerebral artery occlusion (MCAO) for 1 h followed by reperfusion for 23 h.

**Results:**

ND02 treatment of SH-SY5Y cell lines blocked TNF-α-induced nuclear factor-κB (NF-κB) and IκB-α phosphorylation and increased Akt phosphorylation. LY294002 blocked TNF-α-induced phosphorylation of Akt and reduced the phosphorylation of both IκB-α and NF-κB. At doses higher than 10 mg/kg, ND02 had a significant neuroprotective effect in rats with cerebral ischemia and reperfusion (I/R). ND02 (25 mg/kg) demonstrated significant neuroprotective activity even after delayed administration 1 h, 3 h and 5 h after I/R. ND02, 25 mg/kg, attenuated histopathological damage, decreased cerebral Evans blue extravasation, inhibited NF-κB activation, and enhanced Akt phosphorylation.

**Conclusion:**

These data show that ND02 protects brain against I/R injury with a favorable therapeutic time-window by alleviating cerebral I/R injury and attenuating blood-brain barrier (BBB) breakdown, and that these protective effects may be due to blocking of neuronal inflammatory cascades through an Akt-dependent NF-κB signaling pathway.

## Background

Ischemic brain injury resulting from diseases such as stroke is the third leading cause of death in the United States and a leading cause of lethality and disability in European countries. Although much is now known about the molecular consequences of ischemic brain injury, few therapeutic treatments have proven successful in clinical trials [1]. The inflammatory response to brain injury plays a vital role in the pathogenesis of stroke [2,3]. Previous studies have demonstrated that agents with anti-inflammatory action have therapeutic potential for experimental stroke [4].

There is ample evidence indicating that NF-κB is activated in cerebral ischemia and reperfusion (I/R), especially in neurons [5-8]. This suggests that inhibition of NF-κB may represent a treatment strategy in ischemic stroke. Previous studies have shown that aspirin and several structurally diverse compounds provide neuroprotection during cerebral ischemia via inhibition of NF-κB activation [9-13].

Akt, a serine/threonine protein kinase, plays a critical role in controlling the balance between apoptosis and cell survival in response to extra- and intracellular signaling. Pathological mechanisms after cerebral ischemia involve phosphoinositide 3-kinase (PI3K)/Akt [14-17]. Akt signaling exerts its neuroprotective role in cerebral ischemia animal models [18-20] via blocking of NF-κB activation, through IkB phosphorylation and degradation [21].

**Figure 1:**
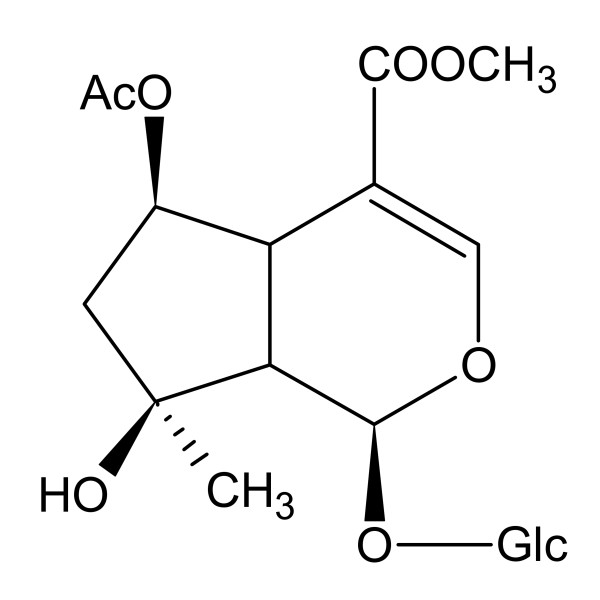
**The chemical structure of ND02**.

6-O-acetyl shanzhiside methyl ester (ND02, Figure 1) is an iridoid glucoside compound isolated from the leaves of *Lamiophlomis rotata (Benth.) Kudo*., which is a Chinese folk medicinal plant in Xi-zang (Tibet). For thousands of years, *Lamiophlomis rotata *has been used in pain alleviation, detumescence, hemostasis, marrow reinforcement and promotion of blood circulation to remove stasis [22]. There are many iridoid glucoside compounds in the leaves of *Lamiophlomis rotata (Benth.) Kudo*, however, ND02 and 8-O-acetyl shanzhiside methyl ester are the main components. Previous studies have shown that 8-O-acetyl shanzhiside methyl ester attenuates apoptosis and ameliorates mitochondrial energy metabolism in rat cortical neurons exposed to oxygen-glucose deprivation [23]. ND02 is also found in the root of *Phlomis medicinalis Die *[24]. The objective of this study was to determine the effects of ND02 on the inflammatory response of neurons and to explore whether ND02 can protect brain against injury in a rat model of I/R.

## Methods

### Materials

ND02 (Molecular Formula: C_19_H_28_O_12_; CAS NO.: 110186-13-5; Chemical name: methyl(1 S,4aS,5R,7 S,7aS)-5-acetyloxy-7-hydroxy-7-methyl-1-[(2 S,3R,4 S,5 S,6R)-3,4,5-trihydroxy-6-(hydroxymethyl)oxan-2-yl]oxy-4a,5,6,7a-tetrahydro-1H-cyclopenta [c] pyran-4-carboxylate, purity > 98.5%) was provided by the State Key Laboratory of Long-acting Extended-Release and Targeting Drug Delivery Systems (Luye Pharma Group Ltd.), Yantai, P. R. China). Its extraction and isolation were carried out according to a previously described procedure [24].

### Cell culture

Human neuroblastoma (SH-SY5Y) cells were obtained from the Shanghai cell bank of the Chinese Academy of Sciences. SH-SY5Y cells were cultured and maintained in F12 + DMEM (1,1, v/v) media, supplemented with 10% FBS and 1% penicillin/streptomycin. Cells were kept at 37°C in a humidified 5% CO_2_/95% air incubator. For TNF-α-stimulated SH-SY5Y cell line experiments in vitro, SH-SY5Y cells (5 × 10^6^) were pre-incubated with PI3 kinase inhibitor, LY294002 (30 μM, Calbiochem, La Jolla, CA) or ND02 (20 μM or 40 μM) for 30 min and then incubated with TNF-α (20 ng/ml) for 15 min.

### Western blot analysis

Cells were cultured for 24 h, then washed twice with ice cold PBS on ice and lysed in NP40 lysis buffer (Biosource, Camarillo, CA, USA) (50 mM Tris, pH 7.4, 250 mM NaCl, 5 mM EDTA, 50 mM NaF, 1 mM Na_3_VO_4_, 1% NP-40 and 0.02% NaN_3_) supplemented with 1 mM PMSF and 1 × protease inhibitor cocktail (Sigma, Saint Louis, MO, USA). Equal amounts of cell protein (50 μg) were separated by SDS-PAGE and analyzed by western blot using specific antibodies to phospho-IκB-α, phospho-NF-κB, phospho-Akt and β-actin (as a loading control). The antibodies were all purchased from Beijing Biosynthesis Biotechnology Company (Beijing, P. R. China). Optical densities of the bands were scanned and quantified with a Gel Doc 2000 (Bio-Rad Laboratories (UK) Ltd). Data were normalized against those of the corresponding β-actin bands. Results were expressed as fold increase over control.

### Animals

Adult male Sprague-Dawley rats were obtained from Shandong Luye-Pharmaceutical Company (Yantai, P. R. China). All animals were housed individually at 22 ± 2°C and a relative humidity of 50 ± 10% and with a 12-h light/dark cycle, and had free access to chow and water. The experimental procedures were approved by the Binzhou Medical University's Administrative Panel on Laboratory Animal Care.

### Rat cerebral ischemia study protocol

The body weight of the rats at the time of the experiments was 280-310 g. After 1 week of acclimatization, rats were anesthetized with chloral hydrate (350 mg/kg, *i.p*.). Rectal temperatures were recorded and maintained at 37°C during surgery with a heating-pad. The middle cerebral artery occlusion (MCAO) operation was carried out according to a previous procedure with minor modifications [25]. The left common carotid artery was occluded, and the branches of the external carotid artery were dissected and divided. The internal carotid artery was followed rostrally and a 4-0 filament (Beijing Shadong Biology Company, P. R. China; the diameter of the filament is 0.25, but the diameter of the tip is 0.34 mm to create a globular stopper) was introduced into the internal carotid artery and advanced until resistance was felt. The filament was removed after 1 h. The rats were kept under 24°C-25°C conditions for the first 24 h after surgery.

For dose-response studies, 105 rats were randomly divided into 7 groups of 15 rats each. ND02 at doses of 1.6, 4, 10, 25 and 62.5 mg/kg was administered as a bolus injection into the tail vein 30 min after reperfusion. (The ND02 was dissolved in sterile saline to make stock solutions, and dilutions were then prepared for different final concentrations.) Sham or vehicle-treated rats were injected with saline. Rectal temperature was determined once every 3 h for a total of 8 times. Neurological deficits were determined 24 h after ischemia, followed by brain infarct volume examinations.

For therapeutic time-window studies, 90 rats were randomly divided into 6 groups. ND02 at a dose of 25 mg/kg was administered as a bolus injection into the tail vein at 1 h, 3 h, 5 h and 7 h after reperfusion. Vehicle-treated rats were injected with saline. Neurological deficits were determined 24 h after ischemia, followed by brain infarct examinations.

For anti-inflammatory mechanism studies, 90 rats were randomly divided into three groups, and each of these into three subgroups of 10 rats each. ND02 at a dose of 25 mg/kg was administered as a bolus injection into the tail vein 30 min after reperfusion. Sham or vehicle-treated rats were injected with saline. The three groups were evaluated for Evans blue extravasation, by western blots, and for histopathological damage by NeuN staining 24 h after ischemia.

For long-term studies, 40 rats were randomly divided into two groups of 20 rats each: an ND02-treated (25 mg/kg) group and a vehicle-treated group. ND02 was administered as a bolus injection into the tail vein 30 min after reperfusion and once every 24 h for 7 consecutive days. Vehicle-treated rats were injected with saline. Neurological deficits were determined on the 3rd, 7th and 14th days after I/R. Fourteen days after I/R, 8 rats in each group were randomly selected for brain infarct examination.

### Evaluation of neurological deficits

Neurological deficits were evaluated using a modified six-point scoring method [26] by an investigator who was blinded to the experimental treatment groups. The scale is 0: no neurological deficit (normal); 1: failure to extend the left forepaw fully (mild); 2: circling to the left (moderate); 3: falling to the left (severe); 4: no spontaneous walking with a depressed level of consciousness (very severe) and 5: dead.

### Evaluation of infarct volume and brain water content

Rats were decapitated 23 h after reperfusion, and their brains quickly removed. Total wet weight of the brain was measured accurately, and each brain was then sliced into five coronal sections of 2-mm thickness each and stained with a 2% solution of tetrazolium chloride (TTC, Sigma) in saline at 37°C for 20 min, and then photographed. The images were digitized and used to calculate infarct volume with a Compix system computer (C imaging 1280 system),. Afterwards, brain water content was determined as an indicator of cerebral edema using a wet/dry method as previously described [27].

### Evaluation of blood-brain barrier (BBB) leakage with Evans blue extravasation

Determination of Evans blue extravasation was based on a previous method [28] with minor modifications. After reperfusion, 0.1 ml of 4% Evans blue (Urchem, Shanghai, P. R. China) in 0.9% saline was intravenously administered. Twenty-three hours after I/R, each rat was perfused with 20 ml 10 U/ml heparinized saline to wash out blood. The brain was then isolated, weighed and homogenized in 50% solution of trichloroacetic acid. After centrifugation at 400 × g for 20 min, the supernatant was spectrophotometrically measured at 610 nm. Cerebral Evans blue was quantified as micrograms of dye per gram of wet weight.

### Western blots analysis in I/R rats

For western blot analysis of collected brain tissues, the tissues were defrosted and immersed in ice-cold lysis buffer (50 mM Tris-HCl, 1 mM EDTA, 1 mM EGTA, 0.5 mM Na_3_VO_4_, 0.1% 2-mercaptoethanol, 1% Triton X-100, 50 mM NaF, 5 mM sodium pyrophosphate, 10 mM sodium β-glyceropyrophosphate, 0.1 mM phenylmethanesulfonyl fluoride, and protease inhibitor mixture) for 10 min. Supernatant was collected after a shake in 1% *p*-nitorphenyl phosphate for 10 s, and centrifugation at 4°C and 12,000 × *g *for 30 s. Equal amounts of protein were separated by SDS-PAGE and analyzed by western blot using antibodies (Beijing Biosynthesis Biotechnology Company, P. R. China) to phospho-IκB-α, phospho-NF-κB, phospho-Akt and β-actin. Optical densities of the resultant bands were scanned and quantified with a Gel Doc 2000 (Bio-Rad). The data were normalized against those of the corresponding β-actin bands. Results were expressed as fold increase over sham animals.

### NeuN staining

NeuN-immunolabeling was carried out by incubating free floating coronal sections (5 μm) with anti-NeuN antiserum and 3,3-diaminobenzidine tetra hydrochloride (DAB). First, coronal sections were washed twice for 20 min in TBS buffer (0.15 M NaCl; 0.1 M Tris-HCl, pH 7.5) at room temperature. This was followed by a 30-min incubation in TBS-BSA [TBS containing 1% (w/v) BSA and 3% normal goat serum (NGS)] at room temperature to block nonspecific antibody binding. The sections were then incubated overnight with 1:500 anti-NeuN antiserum (Beijing Biosynthesis Biotechnology Company, P.R. China) diluted in 1% NGS in TBS at 4°C. After three 10-min washings with 1% NGS in TBS, the sections were incubated with 1:250 biotinylated anti-mouse IgG (Secondary antibody, Fuzhong Maixin Biotechnology Company, P.R. China) for 1 h in 1% NGS in TBS at room temperature. NeuN-immunostaining was visualized using the streptomycete antibiotin-peroxidase solution and DAB.

To evaluate neurological damage after cerebral I/R 24 h, the left hemisphere of each brain was cut coronally into three blocks from the level of the optic chiasm and the infundibular stem of the hypophysisi. The middle block was further cut into three sub-blocks. The middle sub-block (0.1 × 0.1 cm) was embedded in paraffin and immunoreacted for NeuN. NeuN-immunopositive cells were counted at 400 × magnification in three selected fields in the central zone of the middle sub-block (0.1 × 0.1 cm). A final value for number of NeuN-immunopositive cells was calculated by averaging the counts of three fields.

To evaluate brain infarct volume after cerebral I/R of 14 days duration, rats were killed 14 days after I/R. The brains were fixed by transcardial perfusion with saline, followed by perfusion and immersion in 4% paraformaldehyde before being embedded in paraffin. Seven coronal sections of tissue were processed and immunoreacted for NeuN for calculation of volume of cerebral infarction [29]. Indirect infarct area was calculated by subtracting the intact area of the ipsilateral hemisphere from the area of the contralateral hemisphere [29]. Infarct volume is presented as a volume percentage of the lesion compared with the contralateral hemisphere.

### Statistical analysis

Neurological deficit scores between groups were analyzed using a nonparametric test. Quantitative data from the experiments are expressed as mean ± SD. Statistical significance was determined by one-way analysis of variance (ANOVA) followed by Dunnett's test. In all cases, differences were considered significant if *P *< 0.05.

## Results

### ND02 blocks NF-κB activation through Akt signaling pathway

**Figure 2:**
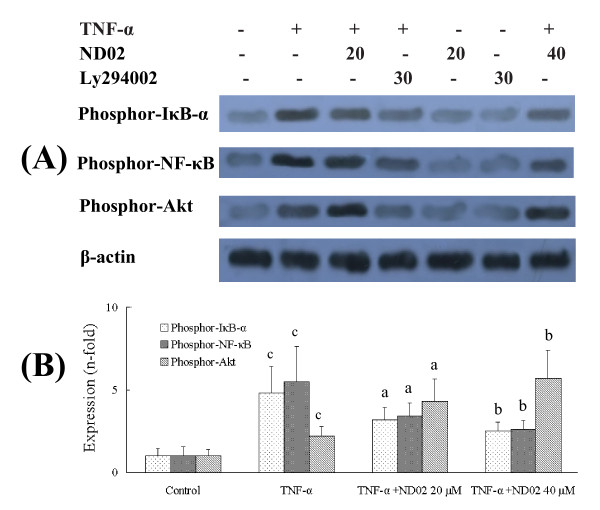
**ND02 inhibits TNF-α-induced phosphorylation of NF-κB and IκB-α through an Akt signaling pathway**. SH-SY5Y cells were pre-incubated with PI3 kinase inhibitor, ly294002 (30 μM) or ND02 (20 μ M or 40 μ M) for 30 min and then incubated with TNF-α (20 ng/ml) for 15 min. Phospho-Akt, Phospho-IκB-α and Phospho-NF-κB expression were analyzed by western blotting. Results are expressed as fold increase over control, n = 5. ^a^*P *< 0.05, ^b^*P *< 0.01 *vs*. TNF-α-induced group. ^c^*P *< 0.01 *vs*. control group. Significance was determined by one-way analysis of ANOVA followed by Dunnett's test.

We compared the effect of ND02 on TNF-α-induced (20 ng/ml for 15 min) activation of IκB-α, NF-κB and Akt in the SH-SY5Y cell line to that of a selective PI3K inhibitor, LY294002. The results (Figure 2) show that pretreatment of SH-SY5Y cells with LY294002 (30 μM) for 30 min blocked TNF-α-induced phosphorylation of Akt and reduced the phosphorylation of both IκB-α and NF-κB. ND02, 20 μM and 40 μM treatments of SH-SY5Y cells, blocked TNF-α-induced IκB-α and NF-κB phosphorylation, while increasing Akt phosphorylation (Figure 2).

### ND02 reduces brain infarct volume, brain water content and neurological deficit scores in cerebral I/R rats

Cerebral I/R leads to severe behavioral disturbance and histological changes in rats. Compared with sham-operated animals, neurological deficit, cerebral infarct volume and cerebral edema were significant higher in cerebral I/R rats.

The results of dose-response studies are shown in Table 1. ND02 at doses of 10, 25 and 62.5 mg/kg, administered intravenously 30 min after I/R, decreased neurological deficit scores, reduced cerebral infarct volume and brain water content in a dose dependent manner.

**Table 1:** Effects of ND02 on survival, neurological scores, infarct volume, and brain water content in ischemia-reperfused rats: a dose-response study

Group	Survival (# rats)	Neurological scores (median/range)	Infarct volume (%)	Brain water content (%)
Sham	15/15	---	---	76.8 ± 0.5
Vehicle-treated	9/15	4/2	27.4 ± 6.3	78.8 ± 0.4
ND02 1.6 mg/kg	9/15	4/3	24.8 ± 4.2	78.6 ± 0.4
ND02 4 mg/kg	10/15	4/3	23.3 ± 4.8	78.4 ± 0.5
ND02 10 mg/kg	10/15	3/3*	19.7 ± 6.2*	78.2 ± 0.7*
ND02 25 mg/kg	11/15	3/4**	17.4 ± 4.8**	77.9 ± 0.5**
ND02 62.5 mg/kg	12/15	2/4**	12.8 ± 3.5**	77.8 ± 0.5**

Data are means ± SD, with n = 15 for each group. ND02, at doses ranging from 1.6 to 62.5 mg/kg, was administered intravenously 30 min after cerebral I/R. ^#^*P *< 0.01 *vs*. sham group; *P < 0.05, **P < 0.01 *vs *vehicle-treated group. Cerebral infarct volume and brain water content between groups were compared using one-way ANOVA followed by Dunnett's test. Neurological scores between groups were compared using a nonparametric test.

The results of therapeutic time-window studies are shown in Table 2. ND02 at a dose of 25 mg/kg significantly decreased neurological deficit scores, reduced cerebral infarct volume and brain water content even with delayed administration 1 h, 3 h and 5 h after I/R. Obviously, earlier administration of this compound brings more therapeutic benefit.

**Table 2:** Effects of ND02 on survival, neurological scores, infarct volume, and brain water content in ischemia-reperfused rats: a therapeutic time-window study

Group		Survival (# rats)	Neurological scores (median/range)	Infacrt volume (%)	Brain water content (%)
Sham		15/15	---	---	76.7 ± 0.5
Vehicle-treated		10/15	4/2	26.1 ± 4.5	78.9 ± 0.4
	1 h	13/15	2/3**	15.8 ± 3.9**	77.9 ± 0.6**
	3 h	12/15	3/4*	18.6 ± 5.1**	78.1 ± 0.4**
ND02 25	5 h	11/15	3/3*	21.8 ± 4.1*	78.4 ± 0.5*
	7 h	10/15	3/3	22.9 ± 6.6	78.6 ± 0.4

Data are means ± SD, with n = 15 for each group. ND02 was administered at a dose of 25 mg/kg at 1 h, 3 h, 5 h and 7 h after cerebral I/R. Data are means ± SD. ^#^*P *< 0.01 *vs*. sham group; *P < 0.05, **P < 0.01 vs vehicle-treated group. Cerebral infarct volumes of different groups were compared using one-way ANOVA followed by Dunnett's test. Neurological deficit scores between groups were compared using a nonparametric test.

**Figure 3:**
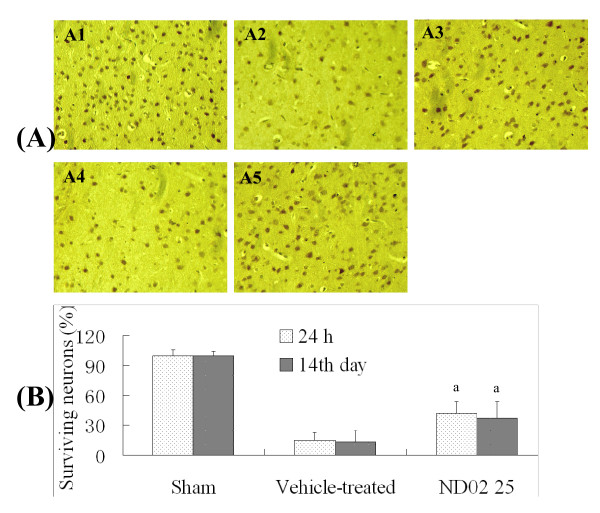
**Effect of ND02 on neurological damage 24 h and 14 days after cerebral ischemia-reperfusion (I/R)**. Figure. 3A: Effect of ND02 on cerebral pathological damage after cerebral I/R. Sections were stained with NeuN after fixation. A1: Sham group; A2: Vehicle-treated group (24 h); A3: ND02 25 mg/kg group; A4: Vehicle-treated group (14th day); A5: ND02, 25 mg/kg, group (14th day). Figure 3B: Effect of ND02 on the NeuN-immunopositive neurons after cerebral I/R. Results were averaged and are expressed as number of NeuN-immunopositive neurons per section. Data are means ± SD, n = 5. ^a^*P *< 0.01 *vs *vehicle-treated group. Significance was determined by one-way ANOVA followed by Dunnett's test.

The results of long-term studies are shown in Table 3. ND02 at an i.v. dose of 25 mg/kg 30 min after I/R significantly decreased neurological deficit scores and reduced cerebral infarction 14 days after I/R. The results of NeuN immunolabeling indicated that this is likely due to a significantly increased number of surviving neurons 24 h and 14 days after cerebral I/R, as shown in Figure 3A and Figure 3B. Thus, it is clear that early treatment with ND02 provides long-term benefits for neuronal functional recovery after cerebral I/R.

**Table 3:** Effects of ND02 on survival, neurological scores, infarct volume, and brain water content in ischemia-reperfused rats: a long-term study

Group	Time (days)	Survival (# rats)	Neurological scores (median/range)	Infarct volume (%)
	3	15/20	4/3	
Vehicle-treated	7	14/20	3/4	
	14	13/20	3/4	27.6 ± 5.8
	3	17/20	2/4**	
ND02 25	7	16/20	2/4*	
	14	16/20	2/4*	13.9 ± 4.7**

ND02 was administered at a dose of 25 mg/kg as a single intravenous bolus injection 30 min after cerebral I/R. This led to significantly decreased neurological deficit scores and reduced cerebral infarct volume within 14 days after I/R. Data are means ± SD, each group is 20 rats. *P < 0.05, **P < 0.01 *vs *vehicle-treated group. Cerebral infarct volumes of different groups were compared using one-way ANOVA followed by Dunnett's test. Neurological deficit scores between groups were compared using a nonparametric test.

### ND02 attenuates the increase of cerebral Evans blue extravasation in I/R rats

Evans blue extravasation was used to assess of BBB breakdown after cerebral ischemia. The results of Evans blue extravasation clearly shows that this pathological consequence of cerebral I/R is significantly attenuated by ND02 treatment, as shown in Table 4.

**Table 4:** Effect of ND02 on cerebral Evans blue extravasation in ischemia-reperfused rats

Group	Dose (mg/kg)	Evans blue extravasation (μg/g wet weight)
Sham	---	0.47 ± 0.11
Vehicle-treated	---	3.31 ± 0.62#
ND02	25	1.76 ± 0.39**

Data are means ± SD, with n = 10 for each group. ^#^*P *< 0.01 *vs*. sham group; *P < 0.05, **P < 0.01 *vs *vehicle-treated animals. Significance was determined by one-way ANOVA followed by Dunnett's test.

### ND02 attenuates the decrease of NeuN-immunopositive neurons

The number of NeuN-immunopositive neuron is a reliable indicator of cerebral injury. After 24 h of IR, the number of NeuN-immunopositive neuron was decreased in cerebral cortex, as shown in Figure 3B. ND02 treatment (25 mg/kg) alleviated this damage to neurons 24 h and 14 days after cerebral I/R.

### ND02 attenuates cerebral NF-κB activation in cerebral I/R rats

**Figure 4:**
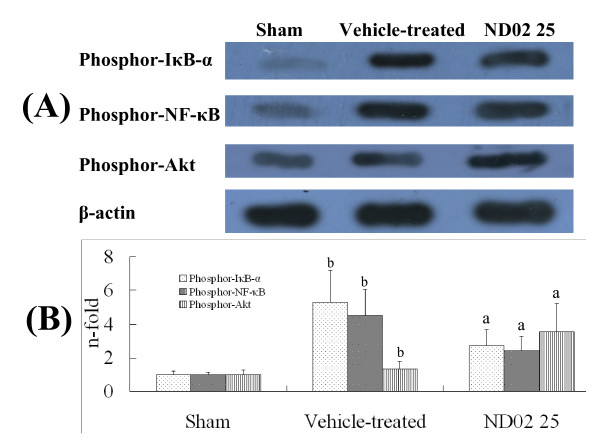
**Effects of ND02 on phosphor-NF-κB, phosphor-IκB-α and phosphor-Akt in cerebral I/R rats by western blots analysis 24 h after I/R**. Rats were subjected to middle cerebral artery occlusion (MCAO) and reperfusion (I/R) for 23 h. Total protein extracts were prepared and assayed for phosphor-NF-κB, phosphor-IκB-α and phosphor-Akt by western blot analysis, and blots were normalized to β-actin expression. Results are expressed as fold increase over sham group, n = 5. ^a^*P *< 0.01 *vs*. vehicle-treated group. ^b^*P *< 0.01 *vs*. sham group. Significance was determined by one-way analysis of ANOVA followed by Dunnett's test.

To investigate the molecular mechanism of the ND02 protective effects, NF-κB activation and phosphorylated Akt were examined. Phosphorylation of IκB-α and NF-κB occurred in rats after cerebral I/R, but were not present in the sham group, as shown in Figure 4. ND02 treatment reduced phosphorylated IκB-α and NF-κB levels, while increasing phosphorylated Akt levels. These data indicate that ND02 inhibits NF-κB activation through Akt signaling pathway in cerebral I/R rats.

## Discussion

In the present study, we observed significant improvement of brain injury after treatment with ND02 in rats subjected to cerebral I/R challenge in an I/R model. Stoke triggers an inflammatory reaction that progresses for hours after the onset of a stroke, and this inflammation plays a central role in the pathogenesis of neuronal injury in ischemic stroke. Inflammation is thought to contribute to the genesis of secondary damage as the consequence of activation of resident perivascular and parenchymal macrophages, and infiltration of peripheral inflammatory cells [30]. This postischemic inflammation occurs a few hours to days after a primary ischemic event, and is associated with delayed injury [31]. In particular, inflammatory reactions contribute to the late stages of ischemic injury and to worsened neurological outcome through multiple mechanisms [32]. The present study indicates that ND02 has anti-inflammatory effects (Table 4, Figure 2 and Figure 4). Treatment with ND02 especially provided long-term benefits for neuronal functional recovery after cerebral I/R (Table 3). This suggests that the neuroprotective effects of ND01 may be to block the inflammatory response. In addition, determinations of ND02 in brain tissue by HPLC after *i.v*. administration of ND02 25 mg/kg indicate that ND02 can penetrate the blood-brain barrier.

Infarction, the main pathophysiological outcome of cerebral ischemia, entails neuronal degeneration and necrosis [33]. Our results (Table 1) reveal that ND02 treatment produces significant reduction in cerebral infarct volume in cerebral I/R model rats. Neuronal degeneration and necrosis have been found to be correlated to deficits in behavioral disturbance, and the present study shows that neurological scores are improved by treatment with ND02 (Table 1). NeuN is a resoluble nuclear protein with a molecular weight of 46 kDa, and is a widely used marker for mature neurons. NeuN is expressed in the nucleus and cell bodies of most neurons but not in glial cells, including oligodendrocytes, astrocytes, and microglial cells [34,35]. Immunoreactivity for NeuN has been reported to decrease dramatically following CNS injury [36-39], and NeuN is a sensitive marker for injured neurons early after ischemic challenge [40]. Thus the number of NeuN-immunopositive neuron in ischemic cerebral cortex is a reliable indicator of cerebral injury. Our data (Figure 3) demonstrate that *i.v*. ND02, 25 mg/kg, attenuates the decrease in NeuN-immunopositive neurons in ischemic cerebral cortex 24 h and 14 days after I/R. This indicates that ND02 is potentially beneficial in treatment of cerebral ischemia.

BBB tight junction permeability is altered in ischemic stroke [41]. BBB breakdown occurs in the early phase (within 24 h) of cerebral ischemia [42,43]. This breakdown of BBB leads to massive brain edema, which is the main cause of death in acute stroke [44]. The brain becomes devoid of protection from toxic substances carried by circulating blood upon BBB disruption. Attenuating BBB disruption provides a strong neuroprotective effect and ameliorates secondary injury [45]. Cerebral Evans blue extravasation is a commonly used biomarker of BBB permeability. Our data (Table 4) demonstrate that ND02, 25 mg/kg *i.v.*, reduces cerebral Evans blue extravasation after cerebral I/R. These results indicate that ND02 improves cerebral I/R injury by attenuating BBB breakdown.

A recent update of the Stroke Therapy Academic Industry Roundtable Preclinical Recommendations [46] lists three potential reasons for failures in translating efficacious preclinical findings into successful clinical trial outcomes: (i) a very tight dose range with sa teep dose-response curve; (ii) inadequate investigation of therapeutic time-windows for initiation of treatment and (iii) differences in observation period between animal models and clinical trials. Consequently, we investigated the dose-response (Table 1), therapeutic time window (Table 2) and long-term efficacy (Table 3) of ND02 in cerebral I/R rats. Our data demonstrate that ND02 exerts potent and long-term neuroprotective effects with an appropriate dose-response curve and a favorable therapeutic time-window in the model of cerebral I/R.

It is well established that NF-κB activation is associated with phosphorylation of IκB-α and NF-κB [47,48]. Animals exempted from NF-κB activation or deficient in NF-κB are less susceptible to cerebral I/R [49]. Akt phosphorylates IκB kinase, and activated IκB-α, in turn, causes activation and nuclear translocation of NF-κB-dependent prosurvival genes [50]. A curb on the NF-κB signaling pathway by ND02 is implicated in its molecular regulation of I/R-triggered inflammatory reactions, because western blot analysis (Figure 4) verified that phosphorylated IκB-α and NF-κB are alleviated, but phosphorylated Akt is increased. Therefore, we believe that the protective effects of ND02 could be due, in large part, to suppression of inflammatory cascades through an Akt-dependent NF-κB signaling pathway.

In summary, the results of the current study suggest that ND02 exhibits significant neuroprotective effects during cerebral I/R injury, including attenuation of BBB breakdown, decreased infarct volume, alleviated cerebral damage, and reduced phosphorylated IκB-α and NF-κB proteins in ischemic brain tissue. These effects of ND02 correlate with inhibition of the inflammatory response. Moreover, unpublished results from our pharmacokinetic laboratory suggest that ND02 might penetrate the blood-brain barrier. These findings point to a therapeutic potential for ND02 as a useful anti-inflammatory lead compound in early cerebral I/R injury.

## Abbreviations

ND02: 6-O-acetyl shanzhiside methyl ester; MCAO: middle cerebral artery occlusion; NF-κB: nuclear transcription factors kappaB; I/R: ischemia and reperfusion; BBB: blood-brain barrier.

## Competing interests

The authors declare that they have no competing interests.

## Authors' contributions

WJ, S Z and F F contributed to the design of the study. H Z contributed to NeuN staining. W J, JH and H Z contributed to operation of animal experiment. All authors read the manuscript, studied it critically for its intellectual content and approved the final draft.
